# The impact of insecticide applications on the dynamics of resistance: The case of four *Aedes aegypti* populations from different Brazilian regions

**DOI:** 10.1371/journal.pntd.0006227

**Published:** 2018-02-12

**Authors:** Gabriela de Azambuja Garcia, Mariana Rocha David, Ademir de Jesus Martins, Rafael Maciel-de-Freitas, Jutta Gerlinde Birggitt Linss, Simone Costa Araújo, José Bento Pereira Lima, Denise Valle

**Affiliations:** 1 Laboratório de Mosquitos Transmissores de Hematozoários, Instituto Oswaldo Cruz, Fundação Oswaldo Cruz, Rio de Janeiro, Brazil; 2 Laboratório de Fisiologia e Controle de Artrópodes Vetores, Instituto Oswaldo Cruz, Fundação Oswaldo Cruz, Rio de Janeiro, Brazil; 3 Instituto Nacional de Ciência e Tecnologia em Entomologia Molecular, Universidade Federal do Rio de Janeiro, Rio de Janeiro, Brazil; 4 Gerência de Controle de Zoonoses, Secretaria Municipal de Saúde, Belo Horizonte/Minas Gerais, Brazil; 5 Laboratório de Biologia Molecular de Flavivírus, Instituto Oswaldo Cruz, Fundação Oswaldo Cruz, Rio de Janeiro, Brazil; Faculty of Science, Mahidol University, THAILAND

## Abstract

**Background:**

In the tropics, the utilization of insecticides is still an important strategy for controlling *Aedes aegypti*, the principle vector of dengue, chikungunya and Zika viruses. However, increasing insecticide resistance in *Ae*. *aegypti* populations might hinder insecticide efficacy on a long-term basis. It will be important to understand the dynamics and evolution of insecticide resistance by assessing its frequency and the mechanisms by which it occurs.

**Methodology/Principal findings:**

The insecticide resistance status of four Brazilian *Ae*. *aegypti* populations was monitored. Quantitative bioassays with the major insecticides employed in the country was performed: the adulticide deltamethrin (a pyrethroid—PY) and the larvicides, temephos (an organophosphate) and diflubenzuron (a chitin synthesis inhibitor). Temephos resistance was detected in all populations although exhibiting a slight decrease over time probably due to the interruption of field use. All vector populations were susceptible to diflubenzuron, recently introduced in the country to control *Ae*. *aegypti*. Resistance against deltamethrin was extremely high in three populations. Molecular assays investigated substitutions in the voltage gated sodium channel (Na_V_), the PY target site, at positions 1011, 1016 and 1534. Elevated frequencies of substitutions Val1016Ile and Phe1534Cys related to high PY resistance levels were identified. Biochemical assays detected alterations in the activities of two detoxifying enzyme classes related to metabolic resistance, glutathion-S-transferases and esterases. The results obtained were evaluated in the context of both recent insecticide use and the records of dengue incidence in each locality.

**Conclusions/Significance:**

The four *Ae*. *aegypti* populations evaluated were resistant to the neurotoxic insecticides, temephos and deltamethrin. However, they were still susceptible to diflubenzuron. A probable correlation between adult insect resistance to PY and the domestic application of insecticides is discussed, pointing to the need for awareness measures regarding the correct utilization by citizens. This work aims to contribute to the efficient and rational management of *Ae*. *aegypti* control of both larvae and adults.

## Author summary

Among the pathogens transmitted by *Aedes aegypti*, dengue virus is the most important due to the number of people affected or at risk and the high rate of mortality worldwide. The confirmation that *Ae*. *aegypti* is also the vector of Zika, chikungunya and urban yellow fever poses serious consequences for public health, pointing to the need of reevaluating current vector control strategies. Although there is growing recognition of the importance of social participation and community engagement to prevent high levels of infestation, insecticides are considered important vector control tools. Nevertheless, the massive and indiscriminate adoption of insecticides to control larvae and adults contributes to resistance spread. In particular, the domestic use of adulticides, especially in epidemic seasons, is assumed to induce high levels of resistance in *Ae*. *aegypti* populations. However, the consequences of insecticide interruption upon the resistance of field populations has been less investigated. We evaluated, in four Brazilian regions over one year, the dynamics of dengue vector population resistance to the principal insecticides used in the country. The main resistance mechanisms were also investigated. Data are discussed taking into account the potential relationship among dengue outbreaks, public and private chemical control and insecticide resistance.

## Introduction

The mosquito *Aedes aegypti* is the main vector of dengue virus, an arbovirus of major importance worldwide. Among the Americas, Brazil is the country most affected by this pathogen [1], considered hyper-endemic, since the four serotypes, DENV-1 to DENV-4, circulate [2]. Recently, two other arboviruses transmitted by *Ae*. *aegypti* have been spreading rapidly, chikungunya (CHIKV) and Zika (ZIKV). CHIK was introduced to America through the Caribbean and its presence was confirmed in Brazil in June 2014 [3]. ZIKV was introduced to the American continent from Northeast Brazil [4]. ZIKV was associated with several neurological disorders, including cases of microcephaly and other developmental alterations in newborns from mothers infected during pregnancy. This suspicion, later confirmed, launched the announcement of a Public Health Emergency of International Concern [5] that persisted until the end of 2016 [6].

Vectorial transmission of arboviruses depends upon three components, the host (in this case, humans), the virus and the vector. Despite intense efforts of biomedical research, when DENV, CHIK and ZIKV are considered, neither effective vaccines for large scale use nor specific drugs, able to block clinical manifestations, are yet available on the market. Thus, strategies that focus on the control of mosquito vectors are currently the main tools against these health problems [1]. From a formal point of view, ‘information, education and social communication’ are a key component of the Brazilian dengue vector control program [7]. However in practice, insecticides play a very important role regarding control actions, from the perspective of both public managers and general society [8].

*Ae*. *aegypti* control in Brazil employs insecticides against larvae and adults. Larvicides are applied in households, 4 to 6 times a year, during visits of control agents, ideally only in water containers that cannot be discarded. In contrast, ultra low volume applications of adulticides do not have a preventive function. In spite of indiscriminate domestic use, these products are employed by health personnel only to block outbreaks in epidemic seasons or at strategic points, aiming to reduce the adult populations. One must be aware that Brazil follows WHO guidelines in order to decide which insecticides are employed in public health. In addition, when larvicides are considered for *Ae*. *aegypti* control, only products approved for drinking water are allowed [9,10].

For a long time, the temephos organophosphate (OP) was the sole larvicide available against *Ae*. *aegypti*. In Brazil, use of this OP started in 1967 when the vector was reintroduced in the country [11]. Due to the dengue epidemic in 1986 [12], its use was intensified [8]. From 2009 on, the spread of temephos resistant *Ae*. *aegypti* populations relegated this OP to a secondary choice of larvicide in the country [13], the recommendation, by WHO Pesticide Evaluation Scheme (WHOPES), of the availability of other products for use in drinking water containers [10], also contributing to this decision. In 2009, the substitution of OP larvicides by Insect Growth Regulators (IGR) began. The first IGR adopted on a national scale was diflubenzuron, a chitin synthesis inhibitor (CSI) [14,15]. Ultimately, it was agreed that a rotation scheme, in principle every four years, would be adopted for larvicides [16].

Until 2001, in addition to the temephos larvicide function, other OPs were used in conjunction for the control of adults. The above mentioned resistance of larvae to temephos induced the adoption of a different approach, consisting of distinct class insecticides against both larvae and adults. The aim in this case was to delay resistance development by varying selection pressure, now exerted with different products in distinct stages of the mosquito life cycle. With this strategy the Brazilian Ministry of Health (MoH) expected to preserve the few products that were still effective. That year, the OPs were replaced by pyrethroids (PY) for adult control. However, a rapid spread of *Ae*. *aegypti* resistance to PY ensued, due mainly to mutations in the target site, the Na_V_ [17, 18]. Therefore since 2009, the MoH initiated the implementation of the OP malathion, the only non-PY adulticide recommended by WHO [9].

Brazil is a country of continental dimensions and, despite general MoH guidelines, there are local decisions, deriving both from public managers and the private initiative, that exert distinct pressures on vector populations. In addition, taking into account the varied genetic backgrounds of *Ae*. *aegypti* from different locations, it is appropriate to assume that distinct resistance profiles and mechanisms can be selected in different geographic regions. This multiplicity of scenarios justifies the importance of monitoring the insecticide resistance dynamics of natural vector populations, whether they are exposed to the pressure of a given insecticide or to its interruption in the field.

In the present study, we evaluated the dynamics of resistance of *Ae*. *aegypti* populations of four distinct Brazilian regions over the course of one year. The chief insecticides employed by the National Dengue Control Program (PNCD) were considered. Two of the major associated mechanisms, metabolic and target site resistance, were also investigated. In the first case, the activity of different classes of detoxifying enzymes was quantified. The target site for PY was analyzed with molecular assays. Several insecticide resistance mechanisms are potentially effective against distinct insecticides simultaneously, belonging or not to the same class, a phenomenon known as cross-resistance. Therefore, the identification of resistance mechanisms involved in each specific situation, together with the possibility of using alternative insecticides bearing different modes of action (such as the use of a CSI to replace OP) is important in vector control programs. Furthermore, comparison of insecticide resistance levels and resistance mechanisms with the local history of chemical control can contribute to a rational management of resistance and, consequently, preservation of the insecticides still available.

## Materials and methods

### Ethics statements

The use of anesthetized mice to blood feed mosquitoes was authorized by Fiocruz Ethical Committee for Animal Use (CEUA P-0498/08 and CEUA L-0007/09)

### Study areas

**Fig 1:**
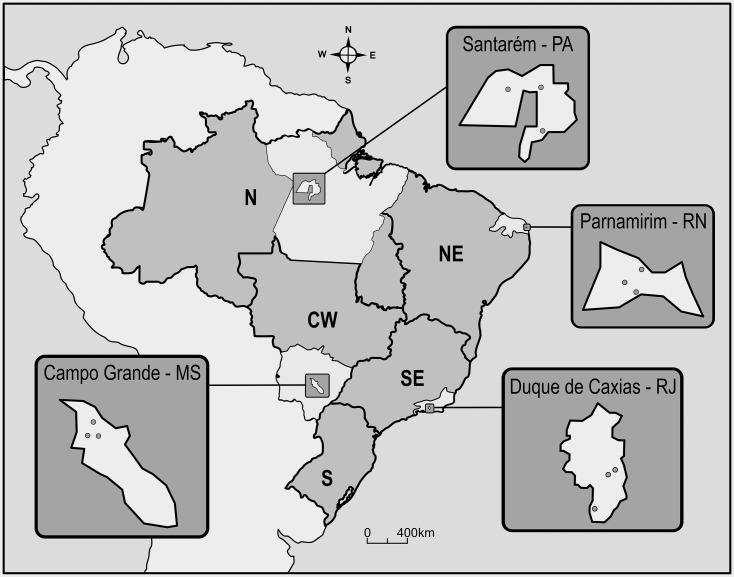
Map of Brazil showing the location of the four municipalities evaluated. In the course of one year, in each municipality, *Ae*. *aegypti* eggs were gathered in the field monthly, in three 1 km^2^ areas, as indicated by grey circles. Letters beside city names account for the respective States: Santarém—Pará (PA), Parnamirim—Rio Grande do Norte (RN), Duque de Caxias—Rio de Janeiro (RJ) and Campo Grande—Mato Grosso do Sul (MS). References to the different Brazilian Regions are placed directly on the map: N—North, NE—Northeast, SE—Southeast, S—South, CW—Central-West.

Dengue is endemic throughout Brazil, except for the southernmost region. Four midsized tropical cities, each one in a different region, all with representative climate regimes and significant chronicles of dengue cases, were chosen (Fig 1, more details in [19]):

Santarém, Pará (PA) State, North Region. 2°26’35”S, 54°42’29”W. This city, located in the Amazon region, bears an elevated humidity, high precipitation indices and temperatures ranging from 23 to 33°C. Santarém has a demographic density of 12.9 inhabitants/km^2^ [20].

Parnamirim, Rio Grande do Norte (RN) State, Northeast Region. 5°54’56”S,35°15’46”W. Parnamirim has a milder and dryer climate compared to Santarém. Temperatures vary from 22 to 29°C and the city has 1,858 inhabitants /km^2^ [21].

Duque de Caxias, Rio de Janeiro (RJ) State, Southeast Region. 22°47’08”S, 43°18’42”W. Similar to Parnamirim, it is a densely populated city, with 1,828 inhabitants/ km^2^ [22]. Temperature differences among seasons are more pronounced compared to the North and Northeast Regions. During summer, heavy precipitation and flooding often occur.

Campo Grande, Mato Grosso do Sul (MS) State, Central-West Region. 20°26’34”S, 54°38’47”W. Of all cities in this study, Campo Grande is located most above sea level, roughly at 600 meters altitude. Among the four study areas here presented, the highest temperature and precipitation amplitudes were registered in Campo Grande. Winter is particularly dry and cold in this city, with a demographic density of 97 inhabitants/ km^2^ [23].

### Field collection of eggs and *Ae*. *aegypti* samples to establish colonies

Ovitraps were used for monthly egg collection [24], in three 1 km^2^ areas in each of the four municipalities (Fig 1). Egg collection was initiated in November 2009 (Duque de Caxias), December 2009 (Parnamirim and Campo Grande) and March 2010 (Santarém) proceeding for 12 months. In each 1 km^2^ area, 120 ovitraps were installed. In the laboratory, eggs of the parental generation collected in the field were reared until the adult stage, and specimens were identified up to the species level. *Ae*. *aegypti* parental adults always corresponded to at least 90% of field samples, and these mosquitoes were used to search for *kdr* mutations (see below). For each municipality, on four occasions roughly at three month intervals, F1 colonies were established in order to perform bioassays and biochemical tests. As depicted in S1 Table, the number of adult females starting the colonies was always over 500. Except for Parnamirim, 18–20 months after the last egg collection, a new field sample was obtained for each locality, and the F1 derived specimens were submitted to a temephos dose-response assay, as indicated below.

### Mosquito rearing

Synchronously reared F1 L3 instar larvae or 1–3 day old adult females were used for bioassays with, respectively, larvicide or adulticide compounds (see below). Rearing was performed essentially as described by Bellinato et al. (2016) [25]. The Rockefeller (“Rock”) strain was adopted both as an internal quality control of all assays and an insecticide susceptible reference lineage [26]. For each insecticide and each field population, as well as for Rockefeller, effective doses (ED_50_ and ED_95_) were obtained by probit analysis with the aid of Polo-PC software [27]. Resistance ratios (RR) were then calculated by dividing the ED of field populations by that of the corresponding Rock. RR_95_ was used to compare all bioassays in accordance with the Brazilian MoH guidelines [28].

### Larvae bioassays

Quantitative bioassays were employed to evaluate the susceptibility status of field *Ae*. *aegypti* populations against the OP temephos and the CSI diflubenzuron. Eight to ten insecticide concentrations, varying from 0.006 to 0.072 mg/L for temephos and 1.0 to 5.5 μg/L for diflubenzuron, were used per assay. For each insecticide concentration in each assay, four replicates, each one with 20 or 10 larvae were exposed to temephos or diflubenzuron, respectively. This corresponds to a total of 640–800 larvae per temephos assay and to 320–400 larvae in the case of diflubenzuron. Each assay was repeated at least three times on different days, mortality varying between 10 and 95% [29, 30]. Results were registered 24 hours after temephos exposure. In the case of diflubenzuron, according to protocols standardized previously [25, 31, 32, 33] the bioassays were followed until adult emergence of all control specimens, not exposed to the CSI.

### Adult bioassays

Quantification of adult resistance to the deltamethrin PY was also performed through dose-response assays, adhering to methodology adapted from the original WHO protocol, with insecticide impregnated papers [34]. Up to 10 different deltamethrin concentrations were used per assay, varying between 2.1 and 109.6 mg/m^2^, depending upon the susceptibility status of the field sample under test. Assays were repeated at least three times on different days. In all cases, three replicates with 15 to 20 adult females each were used.

### Biochemical assays

Quantification of enzyme activities potentially involved in insecticide detoxification was performed in agreement with a standardized biochemical procedure [35, 36]. In all cases, 80 to 120 non-blood-fed young females (up to 24 hours after emergence), stored at -80°C, were individually analyzed. For each female, the following enzyme activities were quantified: glutathione-S-transferase (GST), esterase (EST) and mixed function oxidase (MFO). Three substrates were employed for EST: *α*- and *β*-naphtyl and *ρ*-nitrophenyl acetates, accounting respectively, for activities named *α*-EST, *β*-EST and *ρ*NPA-EST.

According to former protocols, the 99 percentile of the susceptible control strain Rockefeller (p99Rock) was calculated for each enzyme class. Field population data were classified as follows: enzyme activity of any given population was considered unaltered when 0–15% specimens remained beyond p99Rock; values between 15 and 50% and above 50% were classified as altered or highly altered, respectively [35, 36].

### Molecular assays

Allele-specific PCR was applied to investigate the presence of the Ile1011Met, Val1016Ile and Phe1534Cys mutations in the PY target site, Na_V_. Adults of the parental generation derived from monthly field collected eggs were used for evaluation of 1016 allelic frequencies. The other positions, 1011 and 1534, were investigated in the first and last months of the first year interval. In all cases, genomic DNA was extracted from 30 individual adult males of each field sample. If no substitutions were detected at the three positions in all samplings of a population, 30 additional specimens were submitted to evaluation. This was done in order to obtain more accurate measures of *kdr* frequencies. Males were recruited in order to avoid the risk of contamination with spermathecae in the case inseminated females were used. The methodology described elsewhere [37, 38] was followed.

## Results

### Bioassays with larvae: Temephos

**Fig 2:**
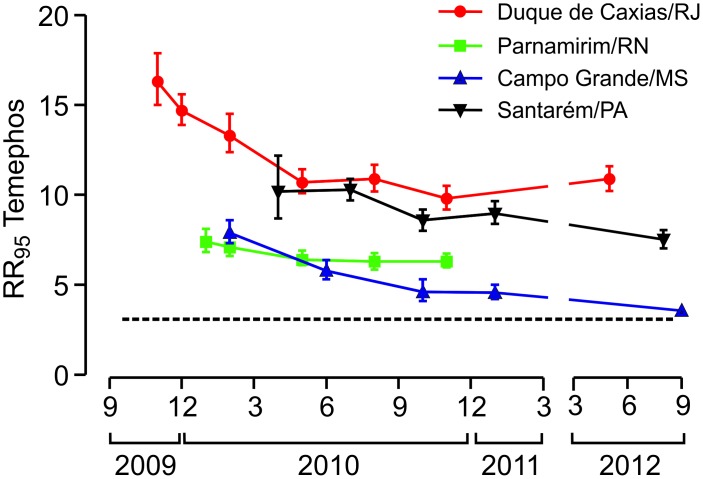
Temporal evaluation of temephos susceptibility status of four Brazilian *Ae*. *aegypti* populations. Each point indicates the Resistance Ratio (RR_95_) and the 95% confidence interval. The dashed line, at RR_95_ = 3.0, points to the threshold that triggers interruption of temephos application, according to the Brazilian Health Ministry [28].

Biological assays identified resistance to temephos in all the *Ae*. *aegypti* populations evaluated throughout the study period (Fig 2, S2 Table). Duque de Caxias presented the highest RR_95_ levels (between 9.8 and 16.3) and Campo Grande, the lowest (3.6 to 7.9). Nevertheless, a temephos resistance decay trend was observed in all cases in the period (2009–2012) although the rate of decay was different among populations. In the course of the study, temephos RR_95_ decreased up to 50% in mosquitoes from Campo Grande, 40% in Duque de Caxias, 30% in Santarém and 15% in Parnamirim. This result is compatible with the withdrawal of temephos in the four municipalities, as reported by each Municipal Health Secretariat. Despite the decrease in temephos resistance, the RR_95_ always remained above 3.0. This value corresponds to the threshold defined by the MoH, above which temephos interruption is recommended [28]. It was also evident that slopes obtained for field populations were always lower than those for Rockefeller (S2 Table), pointing to a higher heterogeneity compared to the control strain.

### Bioassays with larvae: Diflubenzuron

**Fig 3:**
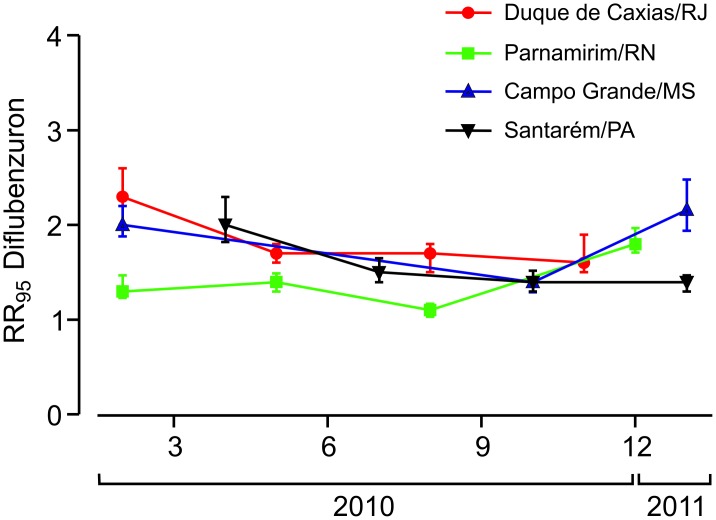
Temporal evaluation of diflubenzuron susceptibility status of four Brazilian *Ae*. *aegypti* populations. Each point indicates the Resistance Ratio (RR_95_) and the 95% confidence interval.

Emergence inhibition of adults (EI) was the parameter evaluated in the dose-response tests of larvae exposed to diflubenzuron. Subtle variations in the effective doses were noted throughout the analyses of all populations with no apparent trend (Fig 3, S3 Table). Diflubenzuron RR_95_ always remained below 3.0 when compared to the Rockefeller strain, indicating susceptibility of field populations to this IGR. Moreover, slopes of the evaluated populations were always higher than the Rockefeller strain (S3 Table), suggesting, unlike results for temephos, a greater homogeneity of these field populations in relation to diflubenzuron susceptibility.

### Bioassays with adults: Deltamethrin

**Fig 4:**
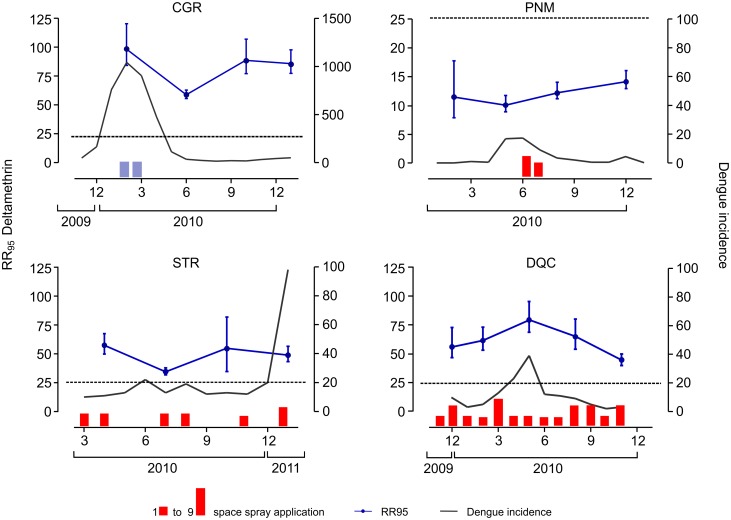
Temporal evaluation of deltamethrin susceptibility/resistance status of adult females from four Brazilian *Ae*. *aegypti* populations: Campo Grande/MS (CGR), Parnamirim/RN (PNM), Santarém/PA (STR) and Duque de Caxias/RJ (DQC). Each point indicates the Resistance Ratio (RR_95_) and the 95% confidence interval. Bars represent the number of deltamethrin (red) and malathion (blue) space spray applications conducted monthly in each municipality, varying between 1 and 9. The dashed line refers to an arbitrary value of 25.0, included in the figure to facilitate visualization of differences in RR among populations (note that y-axis scale, referring to deltamethrin RR_95_, is different for PNM population). Grey lines point to the dengue incidence in the period, expressed in number of cases/100,000 inhabitants. Note that the y-axis scale (Dengue incidence) of the CGR population was altered.

The deltamethrin RR_95_ was extremely elevated in all municipalities, always higher than 10 (S4 Table). Excluding Parnamirim, where deltamethrin RR_95_ ranged between 10.1 and 14.3, all other municipalities were above 35. It is noteworthy that in Campo Grande, for example, the lowest value obtained was 58.2. No trend in RR was noted during the study period, neither a tendency for decrease nor increase. Adult bioassay results are separate for each population, simultaneously with the dengue incidence in the intervals evaluated (Fig 4). In two locations, Duque de Caxias and Campo Grande, the highest RR values were in the period corresponding to the highest dengue incidence. In particular, the numbers of dengue cases in Campo Grande were compatible with an explosive outbreak. Adulticide applications by the municipal health agents in each locality were also included in Fig 4. In this case, only the applications carried out in the study areas (and not in the entire municipality) are shown. Both the intensity and frequency of adult chemical control by the Municipal Health Secretariats varied widely. The intense use of deltamethrin by health agents in Duque de Caxias (much more than the amount recommended by the MoH, see Discussion) should be noted, as well as the use of malathion, a non-PY adulticide, in Campo Grande precisely the municipality where the largest dengue incidence was concomitant with the highest recorded deltamethrin RR_95_. In general, as was the case with temephos and deduced from the slope values, heterogeneity of field populations was higher than that of the Rockefeller strain regarding deltamethrin status.

### Biochemical assays: Metabolic resistance

Adult females were submitted to biochemical assays, disclosing changes in all classes of detoxifying enzymes (Table 1). GST and EST were the most affected activities. However, concerning esterases, greater alterations were observed with the "*ρ*NPA" substrate. The MFO enzymes were the least altered in all populations. Only DQC and PNM populations presented GST and EST altered activities in all evaluated samples. In Campo Grande, no *ρ*NPA-EST alteration was detected, and Santarém was the population with the least changes in the detoxifying enzymes.

**Table 1:** Activity alterations of enzymes related to metabolic resistance in adult specimens of four Brazilian *Ae*. *aegypti* populations.

Population	Period	MFO	α-EST	β-EST	*ρ*NPA-EST	GST
**Duque de Caxias/RJ**	Feb/10		**38**	**17**	**62**	**81**
	May//10	**10**			**57**	**33**
	Aug/10	**21**	**45**	**24**	**22**	**46**
	Nov/10	**15**	**24**	**7**	**26**	**34**
**Parnamirim/RN**	Feb/10		**12**	**6**	**34**	**80**
	May//10	**14**			**43**	**44**
	Aug/10	**14**	**39**	**36**	**7**	**27**
	Dec/10	**5**	**31**	**13**	**23**	**70**
**Campo Grande/MS**	Feb/10	**3**	**5**	**14**	**14**	**76**
	Jun/10	**18**			**7**	**35**
	Oct/10	**3**	**27**	**44**	**1**	**30**
	Jan/11	**10**	**8**	**34**	**4**	**16**
**Santarém/PA**	Abr/10	**7**	**0**	**11**	**11**	**48**
	Jul/10	**31**			**20**	**16**
	Oct/10	**2**	**3**	**15**	**9**	**16**
	Jan/11	**14**	**3**	**9**	**3**	**3**

According to criteria defined previously [35, 36], enzyme activities were classified as unaltered (green), altered (yellow) and extremely altered (pink) if the rate of individuals above the 99 percentile for Rockefeller strain was below 15, between 15 and 50 and above 50%, respectively.

### Molecular assays: The PY target site

**Fig 5:**
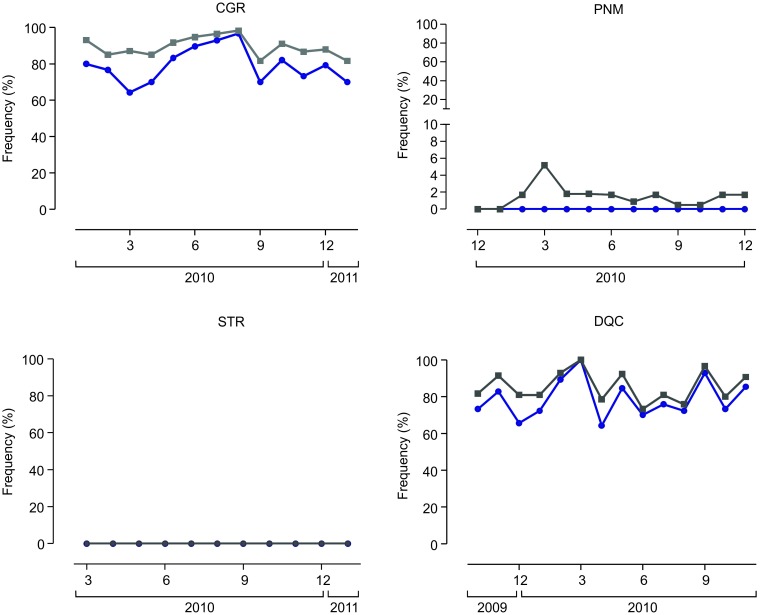
Allelic (grey) and genotypic (blue) frequencies of the *Ae*. *aegypti kdr* Val1016Ile substitution in the Na_V_. Populations names were abbreviated as in Fig 4. Note that the y-axis scale of the Parnamirim (PNM) population was altered in order to enable a better visualization of frequency variations.

The presence and frequency of the Val1016Ile mutation in the *Ae*. *aegypti* Na_*V*_ was investigated monthly in all field populations (Fig 5). Additionally, quantification of two other *Aa*Na_*V*_ mutations, Phe1534Cys and Ile1011Met, was performed in samples collected in the first and last months of evaluation for each population (Table 2). While the relation of the Val1016Ile and Phe1534Cys mutations with PY resistance is well documented [18, 37], the status of the Ile1011Met substitution [39] is still controversial (see Discussion). Substitutions at positions 1016 and 1534 are recessive, i.e., resistance to PY is expressed only in homozygosis [40]. Regarding the third position, there is evidence that the Ile1011Met mutation can be used as a marker of an early duplication event in this species, occurring in the wild type and susceptible genotype. Hence, decrease in the rate of the Ile1011Met substitution should occur in parallel with an increase of the more recent *kdr* mutants in the 1534 and 1016 positions [38].

**Table 2:** Allelic and genotypic frequencies of specific *Ae*. *aegypti* Na_V_ alleles at positions 1016, 1534 and 1011, shown separately, at the first and the last periods of field evaluation.

Population	Period	1016		1534		1011	
		%Ile	%Ile/Ile	%Cys	%Cys/Cys	%Met	%Met/Ile
**Duque de Caxias/RJ**	Feb/10	92.9	89.3	ND	ND	3.3	6.7
	Nov/10	90.7	85.2	98.2	96.4	1.7	3.4
**Parnamirim/RN**	Mar/10	5.2	0.0	33.3	10.0	41.7	83.3
	Dec/10	1.7	0.0	35.0	13.0	43.3	86.7
**Campo Grande/MS**	Mar/10	87.1	64.3	88.0	76.0	13.5	26.9
	Dec/10	87.9	79.3	98.3	96.7	1.8	3.6
**Santarém/PA**	Mar/10	0.0	0.0	94.4	88.9	5.2	10.3
	Jan/11	0.0	0.0	100.0	100.0	0.0	0.0

ND = not determined

Fig 5 shows, for the four populations evaluated, the *kdr* 1016Ile allelic frequency and the genotypic frequency of the *kdr* homozygotes at this position (1016Ile/Ile). For two populations, Duque de Caxias and Campo Grande, rates of the 1016Ile substitution were high throughout the evaluation period, allelic frequencies always above 70%. In these localities, in contrast to the low frequencies in the Ile1011Met mutation, allelic frequencies of the Phe1534Cys substitution were also high (Table 2). The substantial dissemination of these *kdr* mutations in Duque de Caxias and Campo Grande endorsed the high levels of PY resistance previously detected (Fig 5).

In contrast in Parnamirim, mutations at 1016 and 1534 sites are present, notwithstanding at a low frequency. Accordingly in this population, the highest Ile1011Met frequencies were present (Table 2, see Discussion). No homozygous *kdr* specimens at position 1016 were detected in this population (Fig 5, Table 2), and allelic *kdr* 1016Ile rates were always below 10%. Accordingly, deltamethrin RR levels in Parnamirin, although high, were much lower than those observed in the other three populations (Fig 5). Regarding position 1534, approximately 13% of *kdr* homozygotes were found, suggesting participation of the PY target site mechanism in the resistance of this population to deltamethrin.

In spite of the high deltamethrin resistance levels in Santarém (Fig 4, S4 Table), the 1016Ile mutation was not apparent in any mosquito from this population (Fig 5). However, the *kdr* 1534Cys allelic frequencies were high, 94% in the first month of collection and 100% at the end of the work. In parallel, the Ile1011Met frequencies were the lowest, reaching zero in the last evaluation (Table 2).

## Discussion

In Brazil, the monitoring of insecticide resistance in *Ae*. *aegypti* populations assists the rational management of chemical control. We accompanied, over the course of one year, the dynamics of resistance of four field populations belonging to different geographical scenarios and with distinct vector control policies. The quantification of resistance levels together with major resistance mechanisms with respect to the temephos and diflubenzuron larvicides as well as the deltamethrin adulticide, all employed in the control of this vector on a national scale, was considered (S5 Table). The results are discussed taking into account the previous insecticide use and dengue cases in each locality.

In Brazil, since 1967 until recently, temephos was the only larvicide adopted by the public health services for the control of *Ae*. *aegypti*. We confirmed that all populations evaluated in the present study were resistant to this OP. Our results are in accordance with prior reports [13, 25, 41] and even with MoH data which in 2009 already pointed to temephos susceptibility alterations in 90% of the evaluated Brazilian populations [42]. Resistance to OPs has also been observed throughout Latin America, with reports in several countries such as Colombia, Mexico, Cuba, Martinique and Argentina [43–50].

As stated by the Brazilian MoH, recommendations of *Ae*. *aegypti* chemical control management in the country are based on RR_95_ values. In the case of temephos, suspension is indicated when RR_95_ is above 3.0 [28]. In our study, we detected RR_95_ for temephos between 3.6 and 16.3, the highest values in Duque de Caxias and Santarém. Despite the widespread resistance to temephos, in all municipalities a tendency for resistance ratios to decrease was observed during the evaluation period, attributed to the interruption of temephos utilization in the studied areas. However, resistance levels decreased slowly and the temephos RR_95_ of mosquito populations from all localities remained above the susceptibility threshold value, preventing the reutilization of this OP.

Parnamirim, one of the municipalities evaluated here, is located in the metropolitan region of Natal, the capital of RN in the Northeast Region. In accordance with our results, data from the MoH also point to a decrease in *Ae*. *aegypti* temephos resistance levels in Natal, after replacement by *Bti* in 2005. The RR_95_ was 18.6 in 2004 [36] and was reduced to 8.2 in 2007 [28]. In addition, Lima et al. (2011) [51] observed a decrease in temephos resistance in *Ae*. *aegypti* from Juazeiro do Norte, State of Ceará, also in the Northeast Region, after its discontinuation, temephos RR_95_ declining around 30% in six years (from 10.4 in 2003 to 7.4 in 2009). This same work registered an increase of temephos RR_95_ in two locations, Crato and Barbalha, that maintained temephos during this same period, from 7.5 to 30.0 in Barbalha and from 9.0 to 192.7 in Crato. Wirth and Georghiou (1999) [52] also reported, in *Ae*. *aegypti* from Tortola, a small Caribbean island, a decrease in temephos resistance ten years after application interruption in the field. RR_90_, was 46.8 in 1985 [53] and declined to 6.3 in 1995/6 [54].

The *Ae*. *aegypti* resistance status to the inhibitors of chitin synthesis (CSI), another class of larvicides recently introduced in the country, was also quantified. Taking into account the same cutoff established for temephos (RR_95_ = 3.0), diflubenzuron data point to susceptibility of all evaluated populations, confirming previous results obtained in the country for the dengue vector [25, 30, 55]. The recent introduction in Brazil of CSI compounds against *Ae*. *aegypti*, together with their distinct mechanism of action regarding conventional insecticides, contributes to the low resistance rates in the evaluated populations.

In Brazil, except for the State of São Paulo, PY has only been adopted as an adulticide since 2000–2001, after dissemination of resistance to OP in *Ae*. *aegypti* populations was confirmed [56]. This decision was made as a management strategy to expose larva and adult stages to compounds with different action mechanisms. However, mosquito field samples collected shortly afterwards (2002–2003) already exhibited signs of PY resistance [57]. Since then, PY resistance has been detected in several regions of the country [18, 25, 51, 58, 59]. Resistance to deltamethrin was extremely high in all populations studied here. Parnamirim exhibited the lowest RR_95_ levels, despite the magnitude, between 10.1 and 14.3. For the remaining populations, deltamethrin RR_95_ was always above 35.

In Brazil, adulticides are not enlisted by public health managers as infestation prevention tools. Such products are used in attempts to block outbreaks or at strategic points such as airports and other potential vector entry points. According to the MoH, ultralow volume applications of adulticides should not exceed 5–7 times in the course of a year, in general during epidemic periods and in very specific situations and places [7]. However, a survey of the insecticide spatial applications against *Ae*. *aegypti* in the studied localities (Fig 4) revealed a great variation and even an uncontrolled use of these products by local public managers. Many differences were detected among municipalities in adulticide applications, both in frequency and number. In several situations MoH recommendations were far exceeded, and up to nine applications have been registered in one single month. Despite this, the PY resistance levels during the study could not be temporally correlated to the ‘public’ spatial applications of adulticides in each locality. In two populations, Campo Grande and Duque de Caxias, the highest deltamethrin RR_95_ levels were registered precisely during periods of intense dengue transmission. It is worth mentioning that the Brazilian MoH considers that incidence rates beyond 300 dengue cases / 100,000 inhabitants are high [60]. In Campo Grande, in particular, the greatest dengue epidemic ever faced occurred during the period of our study (see Fig 4), when it also presented the highest deltamethrin resistance levels detected throughout the study. However, in this locality deltamethrin was not applied during this outbreak, the adulticide employed being the malathion OP. Our hypothesis is that arbovirus outbreaks cause a collective panic in the local population with a consequent pursuit towards individual protection and control measures. As a result there is a great and uncontrolled rise in the domestic use of PY products that are commercially available. This situation has a direct effect on the elevated PY resistance during epidemic outbreaks [8, 25, 61].

Although the variety of insecticides for public health use is limited, chemical management is still a relevant component of vector control programs. This combination leads to the rapid selection of resistant populations together with the exhaustion of available insecticides, often resulting in control impairment. However, in many situations the suspension of one specific insecticide by a given vector control program does not necessarily lead to its real field interruption due to its intensified domestic use at each new outbreak as well as continued availability of some products in the retail marked. In addition, the lack of integration of the different vector borne disease control programs cannot be neglected.

A significant participation of alterations in the Na_V_, the PY target site in the central nervous system, in the resistance to this class of insecticides was apparent. The Val1016Ile mutation has been previously related to PY resistance in Brazilian *Ae*. *aegypti* populations [18, 37] as well as those of other Latin American countries [62–65]. For this reason its frequency was monitored monthly. Later, the 1534Cys allele was identified in several places throughout the country, even in the absence of the 1016Ile *kdr* allele [37]. We then opted to investigate its frequency together with substitutions in the 1011 position, also potentially interfering with the Na_V_. Besides Brazil, the Phe1534Cys mutation is related to PY resistance in several other localities, such as the Cayman Islands and Thailand [66–67]. In our present study, we always found the 1534Cys mutant allele frequencies higher than those of the 1016Ile *kdr* allele (Table 2), a situation that corroborates previous evidences that the Val1016Ile substitution takes place after the *kdr* mutation at position 1534, in a genetic background already containing the 1534Cys mutation [37, 65, 66, 67]. Regarding the Ile1011Met mutation, there are indications that this substitution can be used as a diagnostic of a Na_V_ duplication event [38] with an unclear relationship with PY resistance. The higher frequencies of 1011Met were evident in populations where the *kdr* 1016Ile and 1534Cys were lower (Table 2).

Duque de Caxias and Campo Grande displayed extremely high levels of resistance to deltamethrin and also very high frequencies of *kdr* mutations at positions 1016 and 1534. In Nova Iguaçu, a municipality contiguous to Duque de Caxias, the 1016Ile mutation was not detected in 2003. However in 2008, the allelic frequency of 1016Ile was 62.5%, two years later peaking at 95% [18, 38]. A rapid increase in the 1016Ile allele was also observed in Campo Grande, frequency of 31.8% in 2008 [18] increasing to values above 85% in 2010 (Table 2), a situation suggestive of a rapid spread of this mutation in the region. The extremely high levels of PY resistance in Duque de Caxias and Campo Grande probably result from the combined effect of both mutations, 1016Ile and 1534Cys, as already described elsewhere [25, 37, 66]. In contrast, as expected, the 1011Met mutation frequencies were low in both municipalities.

In the populations of Duque de Caxias and Campo Grande, the rapid increase in the frequency of the *kdr* allele 1016Ile is indicative of a strong selective pressure. A fast increase in the 1016Ile mutation frequency was also observed in Mexico. Until 1999 this substitution had not been detected, however, it was already high in 2008. In 2011, frequencies above 90% of the 1016Ile allele were reported in three regions of the country, suggesting imminent fixation of this *kdr* allele [68, 69]. There is evidence that identification of PY resistance through laboratory assays may indicate impairment of spatial applications in the field [49]. In addition, similar to what was observed in the field, laboratory selection with PY of six Mexican *Ae*. *aegypti* populations in the course of five generations resulted in the increase of up to three-fold in the frequencies of the 1016Ile allele [70].

Although *Ae*. *aegypti* from Santarém possessed high rates of deltamethrin resistance (RR_95_ between 35.0 and 60.0), the 1016Ile *kdr* mutation was not detected and the 1011Met mutation frequency was very low, reaching zero, in these mosquitoes. Notwithstanding, 90–100% specimens were homozygous for the 1534Cys *kdr* mutation. Parnamirim, in the Northeast Region, presented the lowest deltamethrin RR as well as the lowest *kdr* 1016Ile and 1534Cys frequencies. In particular, the 1016Ile allele remained below 10% throughout the study. It is worth mentioning that mutations in this position had not been detected in NE Brazil in surveys prior to 2010 [18]. Later identification of 1016Ile in mosquitoes from Crato and Juazeiro do Norte, both in the State of Ceará [51], suggests their recent arrival in this Region. The restricted use of PY in the field by Parnamirim local managers, together with the absence of dengue outbreaks in the period with probable reduced domestic use of insecticides, are probably the basis of the comparatively lower PY resistance levels in this locality, as well as their limited variation throughout the study. It is noteworthy that the highest frequencies of the 1011Met mutation appeared in Parnamirim, which is in agreement with previous evidence linking this mutation to a susceptible Na_V_ haplotype [38]. Our results agree with data reported recently, relating high levels of pyrethroid resistance to multiple Na_V_ mutations, a common situation in Latin America *Ae*. *aegypti* populations [71, 72]

Evaluation of metabolic resistance was achieved with biochemical tests quantifying the activity of the main classes of detoxifying enzymes. Although this methodology applied in vector population monitoring routine has the potential to reflect the general dynamics of resistance, we learned with its known limitations that it is not always possible to establish precise correlations between biologic and biochemical assays for each evaluated population at a given moment [36]. Herein, we attempted to compare the metabolic changes of adult females mainly with resistance to the adulticide deltamethrin. As expected, although there was no strict temporal correlation between the levels of PY resistance and the intensity of the metabolic changes for each population, of the three enzyme classes evaluated, GST and EST (and especially ρNPA-EST) were strongly altered while MFO was the least affected class. Regarding MFO, our data, as well as those from other *Ae*. *aegypti* Brazilian populations, differ from other countries whose PY resistance levels tend to correlate with MFO profile alterations [25,36,73]. Still, these results corroborate previous studies that related PY resistance in Brazilian field populations with increased GST and *ρ*NPA-EST activities [36, 74].

Santarém was the population with the lowest contribution of metabolic mechanisms to resistance levels, while Duque de Caxias and Parnamirim were the most affected. Alterations of detoxifying enzymes were identified as the main mechanism of PY resistance in Parnamirim taking into account the low frequency of *kdr* mutations in this population. Different from the other three populations, deltamethrin resistance levels in Parnamirim were consistently lower, also corroborating the strong contribution of PY target site alterations to the intensity of resistance to this class of insecticides, a situation already reported previously [40]. The influence of mutations in the PY target site on elevated resistance levels was confirmed with Santarém mosquitoes, whose high resistance ratios are parallel to the high frequency of the 1534Cys *kdr* allele although the detoxifying enzymes in this population were the least altered in the study. In agreement with this situation, the population of Campo Grande also revealed high levels of PY resistance and high frequencies of *kdr* mutations, while persistent changes in metabolic resistance were only detected for GST enzymes.

Regarding resistance mechanisms, data indicate that different vector populations find different solutions to counteract the challenge represented by insecticides. This is attributed to the multifactorial nature of metabolic resistance as well as the abovementioned limitations of the biochemical methodology employed (which quantifies general activities and not molecular species) [36].

According to Moyes et al. (2017) [72], there is currently plenty of evidence of resistance to the two main classes of insecticides employed all over the world, PY and the OP temephos. In particular, as data related to Latin America abundantly show, high resistance levels are common in the continent. Nonetheless, our results present a trend towards a slow decrease in *Ae*. *aegypti* resistance to temephos since discontinuation of this OP larvicide in the field started in 2009. The CSI susceptible levels are probably a consequence of the recent introduction in the *Ae*. *aegypti* control routine in the country. In contrast, extremely high and disseminated PY resistance levels were noted, indicating a significant participation of the domestic use of this class of compounds in the selection pressure of Brazilian vector populations (S5 Table). The exacerbated domestic use of PY insecticides is seasonal, occurring mainly during outbreaks, and it can be accompanied by the seasonal elevation of resistance levels. Finally, it was possible to highlight the limitations of chemical control as the main methodology for *Ae*. *aegypti* control, taking into account both larvae and adults. There is growing evidence of the need for joint actions with other types of methodologies, social mobilization, mechanical control and biological complementary alternatives. We believe that the adoption of insecticides in a rational way is a strategy to complement other types of controls.

## Supporting information

S1 Table Total of *Ae*. *aegypti* adults obtained after field collection of eggs. The *Ae*. *aegypti* rate related to total *Aedes* specimens and the number of male and female *Ae*. *aegypti* mosquitoes are also shown. Only data from samples used to generate colonies are presented. *Ae*. *aegypti* colonies were employed to evaluate resistance and resistance mechanisms.
(DOC)

S2 Table Details of temephos bioassays with *Ae*. *aegypti* larvae. Results generated by probit analysis.
(DOC)

S3 Table Details of diflubenzuron bioassays with *Ae*. *aegypti* larvae. Results generated by probit analysis.
(DOC)

S4 Table Details of deltamethrin bioassays with *Ae*. *aegypti* adults. Results generated by probit analysis.
(DOC)

S5 Table Temporal evaluation of the susceptibility status of four Brazilian *Ae*. *aegypti* populations to the main insecticides employed by the Brazilian Dengue Control Program. (DOC)
