# Supplementary material for: The impact of insecticide applications on the dynamics of resistance: The case of four Aedes aegypti populations from different Brazilian regions
Source: PLoS Negl Trop Dis. 2018 Feb 12;12(2):e0006227. doi: 10.1371/journal.pntd.0006227 (PMC5833288; doi:10.1371/journal.pntd.0006227)
Supplement: S1 Table — The Ae. aegypti rate related to total Aedes specimens and the number of male and female Ae. aegypti mosquitoes are also shown. Only data from samples used to generate colonies are presented. Ae. aegypti colonies were employed to evaluate resistance and resistance mechanisms. (DOC) [file pntd.0006227.s001.doc]

| **Municipality/State** | **Period** | ***% Ae. aegypti /Aedes*** | **N**  ***Ae. aegypti*** | ♀ | ♂ | **♂/♀** |
| --- | --- | --- | --- | --- | --- | --- |
| **Duque de Caxias/RJ** | Nov/09 | 88 | 5,426 | 2,535 | 2,891 | 1.1 |
|  | Dec/09 | 92 | 2,932 | 1,230 | 1,461 | 1.2 |
|  | Feb/10 | ND | 2,831 | 1,180 | 1,651 | 1.4 |
|  | May/10 | 92 | 9,178 | 4,740 | 3,677 | 0.8 |
|  | Aug/10 | 98 | 2,889 | 1,472 | 1,359 | 0.9 |
| **Parnamirim/RN** | Jan/10 | 99 | 1,293 | 656 | 637 | 1 |
|  | Feb/10 | 98 | 1,541 | 829 | 680 | 0.8 |
|  | May/10 | 99 | 2,228 | 1,386 | 826 | 0.6 |
|  | Aug/10 | 97 | 1,987 | 1,115 | 816 | 0.7 |
|  | Nov/10 | 100 | 1,510 | 764 | 746 | 1 |
| **Campo Grande/MS** | Feb/10 | 99 | 1,399 | 735 | 649 | 0.9 |
|  | Jun/10 | 100 | 933 | 518 | 415 | 0.8 |
|  | Oct/10 | 100 | 1,511 | 896 | 614 | 0.7 |
|  | Jan/11 | 100 | 1,371 | 670 | 696 | 1 |
| **Santarém/PA** | Apr/10 | 100 | 7,486 | 3,628 | 3,858 | 1.1 |
|  | Jul/10 | 100 | 3,708 | 1,824 | 1,884 | 1 |
|  | Aug/10 | 100 | 4159 | 2211 | 1948 | 0.8 |
|  | Oct/10 | 100 | 1,911 | 1,078 | 833 | 0.8 |
|  | Jan/11 | 100 | 5,058 | 2,611 | 2,447 | 0.9 |

ND= not determined
