# Supplementary material for: The impact of insecticide applications on the dynamics of resistance: The case of four Aedes aegypti populations from different Brazilian regions
Source: PLoS Negl Trop Dis. 2018 Feb 12;12(2):e0006227. doi: 10.1371/journal.pntd.0006227 (PMC5833288; doi:10.1371/journal.pntd.0006227)
Supplement: S2 Table — Results generated by probit analysis. (DOC) [file pntd.0006227.s002.doc]

| **population** | **Period** | **LC_50_** | **LC_95_** | **Range** | | | **slope** | | |  |
| --- | --- | --- | --- | --- | --- | --- | --- | --- | --- | --- |
|  |  | **(mg/L)** | **(mg/L)** | **RR_50_** | **RR_95_** | |  |  |  |  |
| **Rockefeller** | **-** | 0.0028 | 0.0053 | 1.0 | 1.0 | | 6.2 | | | |
| **Duque de Caxias/RJ** | Nov-09 | 0.0245 | 0.0865 | 8.7 | 16.3 | | 3.0 | | | |
|  | Dec-09 | 0.0293 | 0.0781 | 10.5 | 14.7 | | 3.8 | | | |
|  | Feb-10 | 0.0245 | 0.0709 | 8.7 | 13.3 | | 3.6 | | | |
|  | May-10 | 0.0236 | 0.0569 | 8.4 | 10.7 | | 4.3 | | | |
|  | Aug-10 | 0.0176 | 0.0580 | 6.3 | 10.9 | | 3.2 | | | |
|  | Nov-10 | 0.0136 | 0.0521 | 4.9 | 9.8 | | 2.8 | | | |
|  | May-12 | 0.0195 | 0.0580 | 6.9 | 10.9 | | 3.5 | | | |
| **Parnamirin/RN** | Jan-10 | 0.0135 | 0.0393 | 4.8 | 7.4 | | 3.5 | | | |
|  | Feb-10 | 0.0159 | 0.0379 | 5.7 | 7.1 | | 4.3 | | | |
|  | May-10 | 0.0143 | 0.0342 | 5.1 | 6.4 | | 4.3 | | | |
|  | Aug-10 | 0.0114 | 0.0334 | 4.1 | 6.3 | | 3.5 | | | |
|  | Dec-10 | 0.0113 | 0.0336 | 3.9 | 6.3 | | 3.5 | | | |
| **Campo Grande/MS** | Feb-10 | 0.0183 | 0.042 | 6.5 | 7.9 | | 4.5 | | | |
|  | Jun-10 | 0.0103 | 0.0308 | 3.7 | 5.8 | | 3.4 | | | |
|  | Oct-10 | 0.0104 | 0.0245 | 3.7 | 4.6 | | 4.4 | | | |
|  | Jan-11 | 0.0093 | 0.0243 | 3.3 | 4.6 | | 3.9 | | | |
|  | Sep-12 | 0.0090 | 0.0190 | 3.2 | 3.6 | | 5.8 | | | |
| **Santarém/PA** | Apr-10 | 0.0200 | 0.0546 | 7.1 | 10.3 | | 5.3 | | | |
|  | Jul-10 | 0.0228 | 0.0548 | 7.9 | 10.3 | | 4.3 | | | |
|  | Oct-10 | 0.0182 | 0.0457 | 6.3 | 8.6 | | 4.1 | | | |
|  | Jan-11 | 0.0190 | 0.0477 | 6.6 | 9.0 | | 4.1 | | | |
|  | Aug-12 | 0.0188 | 0.0400 | 6.7 | 7.5 | | 5.0 | | | |
| LC: lethal concentration; range: 95% confidence interval. | | | | | |  | |  |  | |
|  | | | | | |  | |  |  | |
