# Supplementary material for: The impact of insecticide applications on the dynamics of resistance: The case of four Aedes aegypti populations from different Brazilian regions
Source: PLoS Negl Trop Dis. 2018 Feb 12;12(2):e0006227. doi: 10.1371/journal.pntd.0006227 (PMC5833288; doi:10.1371/journal.pntd.0006227)
Supplement: S3 Table — Results generated by probit analysis. (DOC) [file pntd.0006227.s003.doc]

| **population** | **period** | **LC_50_** | **LC_95_** | **range** | | **slope** |
| --- | --- | --- | --- | --- | --- | --- |
|  |  | **(µg/L)** | **(µg/L)** | **LC_50_** | **LC_95_** |  |
| **Rockefeller** |  | 0.89 | 2.3 | 1.0 | 1.0 | 3.9 |
| **Duque de Caxias/RJ** | Feb-10 | 2.81 | 5.21 | 3.2 | 2.3 | 6.3 |
|  | May-10 | 2.19 | 3.92 | 2.4 | 1.7 | 6.5 |
|  | Aug-10 | 1.98 | 3.77 | 2.2 | 1.7 | 5.8 |
|  | Nov-10 | 2.31 | 3.67 | 2.6 | 1.6 | 8.2 |
| **Parnamirim/RN** | Feb-10 | 1.67 | 3.07 | 2.4 | 1.3 | 6.2 |
|  | May-10 | 1.80 | 3.17 | 2.0 | 1.4 | 6.7 |
|  | Aug-10 | 1.57 | 2.52 | 1.8 | 1.1 | 8.0 |
|  | Dec-10 | 2.57 | 4.19 | 2.9 | 1.8 | 7.7 |
| **Campo Grande/MS** | Feb-10 | 2.44 | 4.69 | 2.7 | 2.0 | 5.8 |
|  | Oct-10 | 1.80 | 3.13 | 2,0 | 1.4 | 6.8 |
|  | Jan-11 | 2.56 | 4.97 | 2.9 | 2.2 | 5.7 |
| **Santarém/PA** | Apr-10 | 2.55 | 4.66 | 2.9 | 2.0 | 6.3 |
|  | Jul- 10 | 1.67 | 3.45 | 1.8 | 1.5 | 5.2 |
|  | Oct-10 | 1.73 | 3.19 | 1.9 | 1.4 | 6.2 |
|  | Jan-11 | 1.79 | 3.25 | 2.0 | 1.4 | 6.4 |
| EI: emergence inhibition; range: 95% confidence interval. | | | | |  |  |
