# Supplementary material for: The impact of insecticide applications on the dynamics of resistance: The case of four Aedes aegypti populations from different Brazilian regions
Source: PLoS Negl Trop Dis. 2018 Feb 12;12(2):e0006227. doi: 10.1371/journal.pntd.0006227 (PMC5833288; doi:10.1371/journal.pntd.0006227)
Supplement: S4 Table — Results generated by probit analysis. (DOC) [file pntd.0006227.s004.doc]

| **population** | **period** | **LC_50_** | **LC_95_** | **range** | | **slope** |
| --- | --- | --- | --- | --- | --- | --- |
|  |  | **(mg/m^2^)** | **(mg/m^2^)** | **RR_50_** | **RR_95_** |  |
| **Rockefeller** |  | 0.70 | 1.45 | 1.0 | 1.0 | 5.0 |
|  | Dec-10 | 12.17 | 82.43 | 17.3 | 56.7 | 2.0 |
| **Duque de Caxias/RJ** | Feb-10 | 21.14 | 89.14 | 30.2 | 61.3 | 2.6 |
|  | May-10 | 22.07 | 115.70 | 31.5 | 79.4 | 2.3 |
|  | Aug-10 | 19.60 | 93.53 | 28.0 | 64.4 | 2.4 |
|  | Dec-10 | 23.45 | 64.87 | 33.5 | 44.5 | 3.7 |
| **Parnamirim/**  **RN** | Feb-10 | 5.04 | 17.01 | 7.2 | 11.6 | 3.1 |
|  | May-10 | 4.41 | 14.75 | 6.3 | 10.1 | 3.1 |
|  | Aug-10 | 6.21 | 18.13 | 8.8 | 12.4 | 3.5 |
|  | Dec-10 | 8.83 | 20.70 | 12.6 | 14.3 | 4.4 |
| **Campo Grande/MS** | Feb-10 | 49.66 | 142.21 | 70.9 | 97.8 | 3.5 |
|  | May-10 | 40.86 | 84.34 | 58.3 | 58.2 | 5.2 |
|  | Oct-10 | 45.12 | 128.17 | 64.4 | 88.3 | 3.6 |
|  | Jan-11 | 42.91 | 124.19 | 61.3 | 85.5 | 3.5 |
| **Santarém/**  **PA** | Apr-10 | 20,00 | 84.17 | 28.5 | 57.8 | 2.6 |
|  | Jul-10 | 19.74 | 51.01 | 28.2 | 35.1 | 3.9 |
|  | Oct-10 | 33.26 | 80.29 | 47.5 | 55.1 | 4.2 |
|  | Jan-11 | 25.81 | 71.73 | 36.8 | 49.3 | 3.7 |
| LC: lethal concentration; range: 95% confidence interval. | | | | |  |  |
