# Supplementary material for: The impact of insecticide applications on the dynamics of resistance: The case of four Aedes aegypti populations from different Brazilian regions
Source: PLoS Negl Trop Dis. 2018 Feb 12;12(2):e0006227. doi: 10.1371/journal.pntd.0006227 (PMC5833288; doi:10.1371/journal.pntd.0006227)
Supplement: S5 Table — (DOC) [file pntd.0006227.s005.doc]

|  |  | **Larvae** | | | | |  | **Adults** | |
| --- | --- | --- | --- | --- | --- | --- | --- | --- | --- |
|  |  | **Temephos** | |  | **diflubenzuron** | |  | **Deltamethrin** | |
| **population** | **period** | **RR_50_** | **RR_95_** |  | **RR_50_** | **RR_95_** |  | **RR_50_** | **RR_95_** |
| **Duque de Caxias/RJ** | Nov-09 | 8.7 | 16.3 |  | ND | ND |  | ND | ND |
|  | Dec-09 | 10.5 | 14.7 |  | ND | ND |  | 17.3 | 56.7 |
|  | Feb-10 | 8.7 | 13.3 |  | 3.2 | 2.3 |  | 30.2 | 61.3 |
|  | May-10 | 8.4 | 10.7 |  | 2.4 | 1.7 |  | 31.5 | 79.4 |
|  | Aug-10 | 6.3 | 10.9 |  | 2.2 | 1.7 |  | 28.0 | 64.4 |
|  | Nov-10 | 4.9 | 9.8 |  | 2.6 | 1.6 |  | 33.5 | 44.5 |
|  | May-12 | 6.9 | 10.9 |  | ND | ND |  | ND | ND |
| **Parnamirin/**  **RN** | Jan-10 | 4.8 | 7.4 |  | ND | ND |  | ND | ND |
|  | Feb-10 | 5.7 | 7.1 |  | 2.4 | 1.3 |  | 7.2 | 11.6 |
|  | May-10 | 5.1 | 6.4 |  | 2.0 | 1.4 |  | 6.3 | 10.1 |
|  | Aug-10 | 4.1 | 6.3 |  | 1.8 | 1.1 |  | 8.8 | 12.4 |
|  | Dec-10 | 3.9 | 6.3 |  | 2.9 | 1.8 |  | 12.6 | 14.3 |
| **Campo Grande/MS** | Feb-10 | 6.5 | 7.9 |  | 2.7 | 2.0 |  | 70.9 | 97.8 |
|  | Jun-10 | 3.7 | 5.8 |  | ND | ND |  | 58.3 | 58.2 |
|  | Oct-10 | 3.7 | 4.6 |  | 2,0 | 1.4 |  | 64.4 | 88.3 |
|  | Jan-11 | 3.3 | 4.6 |  | 2.9 | 2.2 |  | 61.3 | 85.5 |
|  | Sep-12 | 3.2 | 3.6 |  | ND | ND |  | ND | ND |
| **Santarém/**  **PA** | Apr-10 | 7.1 | 10.3 |  | 2.9 | 2.0 |  | 28.5 | 57.8 |
|  | Jul-10 | 7.9 | 10.3 |  | 1.8 | 1.5 |  | 28.2 | 35.1 |
|  | Oct-10 | 6.3 | 8.6 |  | 1.9 | 1.4 |  | 47.5 | 55.1 |
|  | Jan-11 | 6.6 | 9.0 |  | 2.0 | 1.4 |  | 36.8 | 49.3 |
|  | Aug-2012 | 6.7 | 7.5 |  | ND | ND |  | ND | ND |

ND = not determined.
